# Can Pets Prevent Suicide? The Impact of Companion Animals on Suicidality: Scoping Review and Clinical Recommendations

**DOI:** 10.3390/healthcare13233067

**Published:** 2025-11-26

**Authors:** Hannah Van Buiten, Christy Turner, Jaci Gandenberger, Jenni Forkin, Ashley Taeckens, Kevin N. Morris, Leanne O. Nieforth

**Affiliations:** 1 VA Sierra Nevada Health Care, 975 Kirman Avenue, Reno, NV 89502, USA; christy.turner2@va.gov; 2 Graduate School of Social Work, University of Denver, 2142 South High Steet, Denver, CO 80210, USA; jaci.gandenberger@du.edu (J.G.); jenni.forkin@du.edu (J.F.); kevin.morris@du.edu (K.N.M.); 3 Hope Squad, P.O. Box 738116, Dallas, TX 75373, USA; ashley@hopesquad.com; 4 College of Veterinary Medicine, Purdue University, 625 Harrison Street, West Lafayette, IN 47907, USA

**Keywords:** suicide, suicidality, mental health, companion animals, human–animal interactions, pets

## Abstract

**Highlights:**

**What is the main finding?**

- Companion animals have been found to impact suicide risk.

**What are the implications of the main findings?**

- Suicide prevention strategies may benefit from the incorporation of companion animals.
- Future research is needed to better understand the connection between companion animals and suicide risk.

**Abstract:**

Objective: Suicide is widely recognized as an international health epidemic despite significant efforts for prevention. Various psychosocial factors can impact an individual’s suicide risk. This scoping review explored the current literature on the impact of companion animals (i.e., pets) on suicidality. Methods: A scoping review was completed utilizing PRISMA guidelines and the PRISMA Scoping Review Extension to assess the scope of existing research on this topic. The extracted studies were reviewed by a team of research and mental health professionals to develop themes and clinical recommendations, ensuring a direct bridge from research findings to practical application. Results: The search yielded 1948 articles for title and abstract review, of which 64 were retained for full-text review, with 30 meeting the inclusion criteria. Included studies were reviewed by the research team, and data were extracted and discussed. Themes were identified as they relate to suicidality and summarized as representing protective factors against suicide, risk factors for suicide, unclear/inconclusive impact on suicidality, or no impact on suicidality. Conclusion: The results of this review provide insight into the complex ways in which pets can contribute to both protective and risk factors for suicidality. Understanding this impact can inform clinical practice and suicide prevention efforts.

## 1. Introduction

Suicide is recognized as an international health epidemic by the World Health Organization [1], with as many as 720,000 people dying globally as a result of suicide each year. According to the Centers for Disease Control and Prevention (CDC), suicide is one of the leading causes of death in the United States (U.S.), with 49,476 individuals dying by suicide in 2022 [2]. In further data, the U.S. Substance Abuse and Mental Health Services Administration (SAMHSA) reports that 13.2 million adults seriously considered suicide, 3.8 million adults made a suicide plan, and 1.6 million adults made a suicide attempt in 2022 [3]. Research has demonstrated that suicide rates are impacted by a variety of factors that include race, ethnicity, age, sex, and other demographics. Annually, a disproportionate number of people of color die by suicide, with the highest rates experienced by Non-Hispanic American Indian/Alaska Natives populations [2]. The CDC has additionally reported that males are more likely than females to die by suicide, with males making up as many as 80% of suicides in 2022 despite representing approximately half of the U.S. population. Additional demographics, including military Veterans, are disproportionately impacted by suicide. According to the Department of Veterans Affairs (VA) Office of Mental Health and Suicide Prevention, suicide was the 12th leading cause of death for U.S. military Veterans in 2022 [4]. This equates to 17.5 Veterans dying of suicide per day, approximately double the rate of U.S. civilians.

Given the scope and severity of this problem, identifying effective interventions for the prevention of suicidality (i.e., feelings, behaviors, and thoughts about ending one’s life) and death by suicide is a timely and important area of growing research. Many treatments, interventions, and initiatives have shown efficacy in the treatment of mental health diagnoses and suicide prevention. Yet, despite the development of these interventions, data indicate that deaths by suicide amongst adults in the U.S. have risen from 2001 to 2021 [5], highlighting a critical need for continued development of prevention and intervention efforts.

Protective factors which reduce suicide risk are varied and representative of psychological, behavioral, and social factors. For example, protective factors include life satisfaction, social connectedness, and a sense of belonging for older adults [6] as well as high-quality relationships with family, positive connections with peers, self-esteem, emotional intelligence, and utilization of coping skills for adolescents [7]. Societal level factors (e.g., poverty), individual-level factors (e.g., problem solving skills, self-efficacy, hopefulness), psychosocial-level factors (e.g., social support, being married, supportive school environments), and positive reasons for living (e.g., personal relationships, children, spirituality) all impact the likelihood of suicidal behaviors [8]. Multiple psychological frameworks have been developed in an effort to understand suicidality to include the Interpersonal Theory of Suicide [9,10] which hypothesizes that suicide risk is impacted by thwarted belongingness (i.e., a sense of not feeling connected to others) and perceived burdensomeness (i.e., a belief that one is a burden to others).

One avenue for suicide prevention may already be present within many U.S. households. Having a companion animal has proven to be impact human health, with documented physical health benefits such as reducing the risk for cardiovascular diseases [11] and improving well-being for people with chronic pain [12]. Having a pet has also been shown to strengthen social connections and improve mental health by alleviating loneliness [13,14] which presents the possibility that having a pet may contribute to known suicide protective factors as discussed above. Further, nearly half of the U.S. population has a pet, with 2021 Census data reporting 49.6% (63.8 million) of American households include at least one companion animal [15]. The “pet effect,” a term coined by anthrozoologists studying the implications of companion animals on human well-being [16], is a growing area of research. For example, research has identified that pets were found to be an integral form of social support for individuals with long-term mental health diagnoses, which was identified as especially true for those with limited supportive relationships with humans [17,18]. Pets have also proven to decrease loneliness in adults [19], older adults [20], and rural adolescents [21]. Given this, pets may contribute to reduced thwarted belongingness, thus impacting suicide risk [10].

### 1.1. Objectives

The primary objective of this scoping review was to determine what is known about the impact of companion animals on suicidality. Specifically, there is a need to combine and summarize the breadth of literature to both clarify the effect of companion animals on suicidality and identify any current gaps where further research is needed. Secondarily, the researchers aimed to understand whether the literature has labeled companion animals as a protective factor, a risk factor, or as having no impact on suicidality.

### 1.2. Research Question

Research team members engaged in thorough discussion to develop the following research question: What is the scope of existing research on the effects of companion animals on human suicide and suicidality?

## 2. Methods and Analysis

The research team utilized the PRISMA Extension for Scoping Reviews as a checklist and guideline to conduct this study [22]. The review protocol was published on OSF Registries on 8 October 2024 (DOI: 10.17605/OSF.IO/69WMP) [23]. The research team agreed on the review’s inclusion criteria through iterative discussion. Articles were deemed suitable if they pertained to (1) human–animal interactions with pet(s) or companion animals and (2) suicidality or suicide; (3) were peer-reviewed; (4) were published in English, Spanish, or French. No restrictions were established for the date of publication, human subjects involved, species of pet, or data type (i.e., quantitative or qualitative) in order to capture all relevant works. The authors also included studies where suicidality was either a primary or secondary outcome. While most grey literature, to include unpublished research, research or government reports, and conference abstracts were not included, dissertations were included as they represent original research and were identified by the research team as being useful in capturing emerging topics and increasing our breadth of evidence. The use of grey literature in reviews articles is supported by Mak and Thomas [24] as well as Nair and Borkar [25] to reduce publication bias. Systematic reviews or scoping reviews were excluded as they do not represent original research. As this study focuses on the impact of companion animals as pets, articles pertaining to service animals and therapy animals were excluded, as these animals are specifically trained to fulfill a different, and intentionally therapeutic, role in the lives of humans. Research pertaining to emotional support animals was included as emotional support animals are companion animals that provide comfort, companionship, and/or emotional stability to a person with a psychological diagnosis without specialized training, and are handled by an individual for their personal benefit (versus a therapy dog which is handled by a therapy professional for goal-directed therapeutic benefit within the context of treatment). As such, the research team determined emotional support animals serve a similar purpose to companion animals kept as pets, which informed the decision to include them in this review. The research team additionally agreed to exclude articles exclusively related to the suicide risk of veterinarians within the context of their occupation. The authors concluded that these articles described an occupational hazard outside the scope of this review. However, articles pertaining to veterinarians’ suicide risk as it relates to a personal, non-occupational relationship with a companion animal were included.

The authors also intentionally excluded criteria related to self-harm outside of the context of suicidality for this scoping review. Since suicide and self-harm are often discussed in relation to each other in the literature, despite a lack of causation (i.e., one who self-harms may not be suicidal; not all who die by suicide self-harm before attempting suicide), articles exclusively related to self-harm were excluded as they were deemed irrelevant to the research question.

### Study Selection

The following search terms were used and adapted for each database: suicid* AND companion animal* OR pet or emotional support animal* OR domesticated animal* OR cat OR cats OR feline OR felines OR dog OR dogs OR canine OR canines. Databases searched included PubMed, Web of Science-Core Collection, EBSCOhost, ProQuest, Scopus, and ScienceDirect. Wildcards, such as asterisks, were used in the search string to widen the search and capture multiple variations of the search terms (e.g., suicid* utilized to capture suicide, suicides, suicidality, suicidal). For databases not accepting wildcards, the following search string was utilized: (suicide OR suicidal) AND (“companion animal” OR pet OR pets OR “emotional support animal” OR cat OR cats OR feline OR dog OR dogs OR canine). When available, language filters were utilized to search for articles in English, French, and Spanish. No date restrictions were utilized in the search. The search string was developed in consultation with a research librarian. Dogs and cats were included in the search string given their prevalence as common companion animals.

Screening was conducted by the research team in stages. First, studies were reviewed by title and abstract utilizing the predetermined inclusion and exclusion criteria to filter relevant articles by a minimum of two researchers, and any conflicts were discussed amongst the research team. Covidence software (Version 2025, Melbourne, Australia) automatically filtered for duplicates [26]. Articles that met inclusion criteria upon title and abstract review, as well as those with insufficient information to determine eligibility, were submitted for full-text review. Full-text review was also completed by a minimum of two researchers, and any conflicts were discussed amongst the full research team. Once studies were selected for inclusion, data extraction was completed by two reviewers, again gaining full team consensus as needed. Using Covidence’s data extraction tool, two independent extractors recorded data from each reference on: (1) Country in which the study was conducted, (2) Language in which the study was published, (3) Study Aim, (4) Study Methods and Design, (5) Participant demographics, (6) Study Inclusion and Exclusion Criteria (7) Pet Ownership Information, (8) Study Outcomes and Limitations. Study outcomes were categorized as one or more of the following: companion animals are (1) a protective factor against suicide, (2) a risk factor for increased suicide risk, (3) have no impact on suicidality, or (4) inconclusive or unclear impact on suicidality. Study outcomes were determined to have an inconclusive or unclear impact on suicidality when they did not explicitly identify ways in which companion animals impacted the suicide risk of their sample. Once data extraction was completed by two independent extractors, consensus was determined by identifying conflicts and developing resolution through discussion amongst the research team. Extracted data were analyzed by the research team and summarized into predominant themes, which are reported in this final manuscript. Statistical analysis was not completed as a part of this scoping review.

## 3. Results

**Figure 1:**
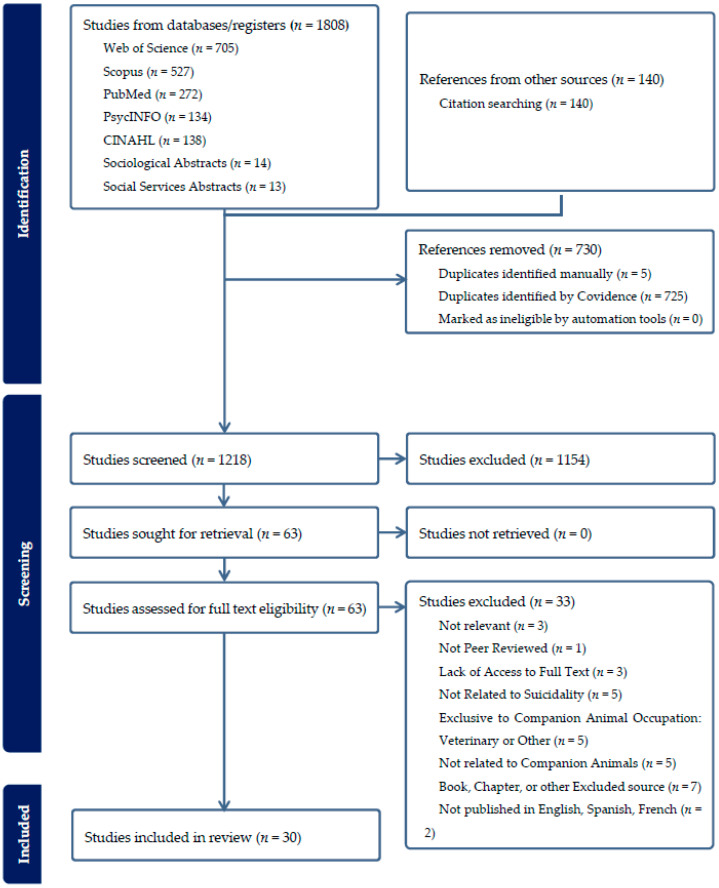
PRISMA Flow Diagram.

The initial database search yielded 1948 results. After removing 730 duplicates, 1218 studies were screened by title and abstract, with 1154 identified as irrelevant. The remaining 64 studies underwent full-text review. Of these, 30 studies were selected for inclusion in this scoping review as represented in Figure 1.

The selected articles employed various scientific approaches. A majority (*n* = 15) of the articles presented qualitative data, while others represented case reports (*n* = 8), cross-sectional studies (*n* = 1), and prevalence studies (*n* = 1). Articles were published between 1985 and 2024, and most (*n* = 29) were published in English, in the U.S. (*n* = 8), the United Kingdom (*n* = 7), or Australia (*n* = 5). Articles were published in journals from various disciplines that included forensic science, psychology, veterinary medicine, anthrozoology, and military studies. Additional information on selected studies is presented in Table A1 (in Appendix A).

Dogs (*n* = 24) and cats (*n* = 15) were the most commonly mentioned species. Fish, horses, small mammals, and birds were also represented (see Table 1). A majority of the research focused on adult populations (*n* = 21) and included individuals experiencing homelessness, LGBTQIA+ individuals, women who have experienced interpersonal violence, older adults, psychiatric patients, adults living with human immunodeficiency virus (HIV), veterinary professionals, Veterans, students, and individuals diagnosed with autism spectrum disorder. Some, but not all, of the selected articles focused on specific mental health diagnoses to include depression, anxiety, bipolar disorder, and more.

### 3.1. Impact on Suicidality

#### 3.1.1. Pets as Protective Factors

**Figure 2:**
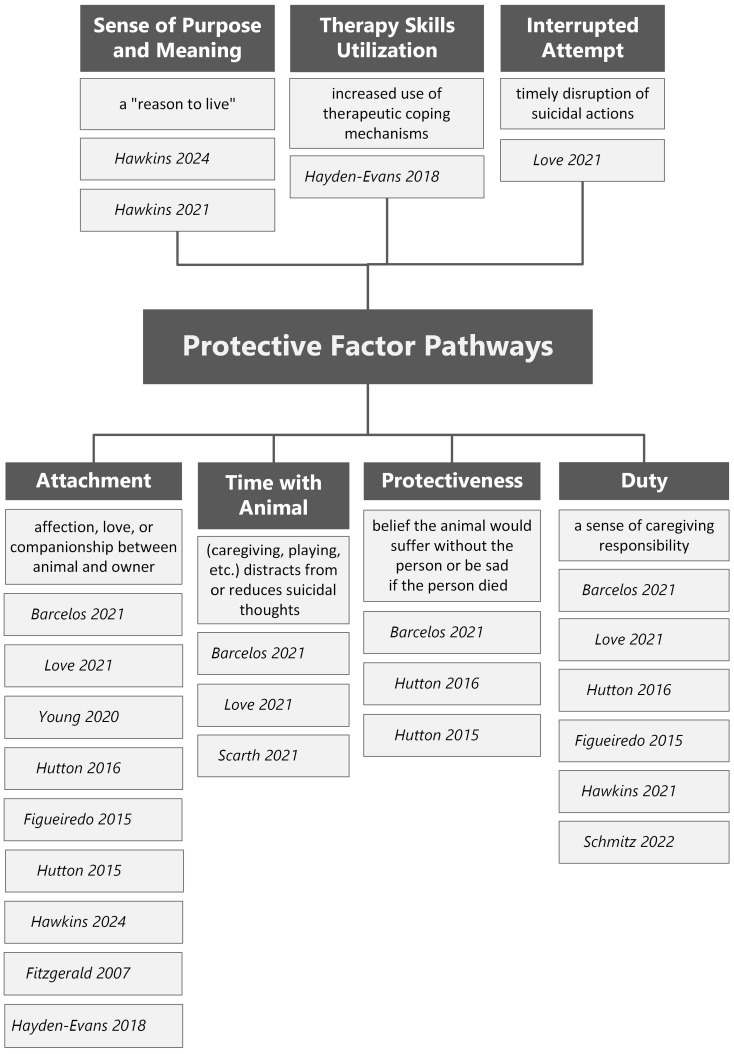
Protective Factor Pathways [27,28,29,30,31,32,33,34,35,36,37,38].

Nine (*n* = 9) articles identified in this review demonstrated the capacity for animals to serve as a protective factor against suicide. Multiple pathways for suicide prevention were represented in the research (see Figure 2). The most frequently identified mechanism for suicide prevention was attachment to a companion animal through affection, love, or companionship [27,28,29,30,31,32,33,34,35]. For example, a suicidal individual living with HIV identified “unconditional love” from their pet as a primary protective factor against suicide [32]. A “sense of connectedness” between participants and their pets [33] as well as participants considering their pets “like family” [34], symbolized the strong emotional bond that can serve as a protective factor against suicide. Attachment to an animal was also shown to exist in the absence of attachments to other people, as is the case in the older adult population studied by Figueiredo et al. [31].

Another identified mechanism for suicide prevention was caregiving responsibility [27,28,30,31,36,37]. The consistent and regular responsibilities of petkeeping provided a reason to live due to a sense that the pet could not survive without their human and that the pet “needed” them [30]. Further, research indicated that a belief the animal would suffer psychologically and/or physically following their death by suicide was a protective factor for multiple individuals [27,30,32]. Companion animals also represented an enhanced sense of purpose and meaning, which created a “reason to live” and served as a buffer against suicidality in two studies [33,36]. Relationships with companion animals additionally seemed to offer necessary distractions from suicidal thoughts [27,28,38]. Among those diagnosed with borderline personality disorder, having relationships with companion animals was also associated with an increase in utilization of psychotherapy tools such as mindfulness; though this hypothesis/conclusion requires further research [35]. In one study [28], a pet dog interrupted a suicide attempt by “crying” when their caretaker attempted to jump from a window, prompting the individual to disengage from the suicidal behavior.

#### 3.1.2. Pets as Risk Factors

**Figure 3:**
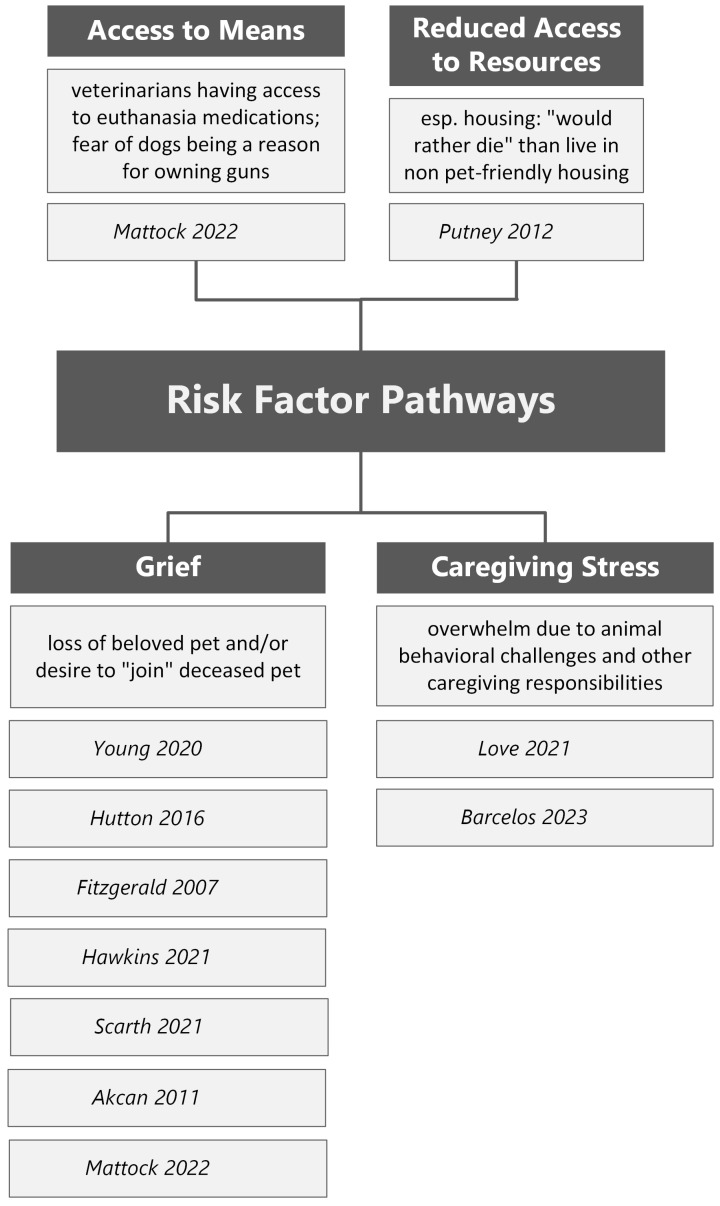
Risk Factor Pathways [28,29,30,34,36,38,39,40,41,42].

The research also revealed that pets can pose a unique risk factor for suicide (see Figure 3). Most notably, grief and loss associated with the death or loss of a companion animal were represented as a risk factor for suicide in multiple studies [29,30,34,36,38,39,40]. The emotional distress associated with the loss of a pet can contribute to suicidal ideation and/or behaviors, with one individual noting they wanted to “join” a beloved deceased pet [40] in their suicide note. Another identified theme was the stress associated with animal caregiving to include animals with behavioral difficulties [28,41]. Caring for an animal with behavioral or medical challenges can contribute to isolation and other concerns, which may impact suicidality [42]. Access to resources was also identified as a challenge for some individuals, such as one participant reporting they would rather die by suicide than live in housing that does not accommodate their pet [42]. Another study represented that personal and/or professional interrelations with animals could indirectly increase access to suicidal means [40]. This study outlined specific examples documented in coroner reports, including an individual who died by suicide utilizing a gun purchased due to a fear of dogs, an individual who used a dog leash as a hanging device, and multiple separate cases in which veterinarians died by suicide using the drugs typically used to euthanize animals in their veterinary practice.

Another potential risk factor for suicide associated with having a companion animal is exposure to *Toxoplasma gondii (T. gondii). T. gondii* is a parasite that can be found in cat feces, among other sources [43]. Exposure to *T. gondii* has been shown in multiple studies [44,45] to correlate with increased suicide risk and suicidal behaviors. While there is little research which directly connects cat ownership, parasite exposure, and increased suicide risk, an article included in our review [46] did identify that individuals who were in contact with cats were more likely to be exposed to *T. gondii*, and those who were exposed to *T. gondii* experienced more suicide attempts among an inpatient psychiatric cohort in Iran.

#### 3.1.3. Pets’ Inconclusive Impact

Ten studies reported inconclusive results related to the impact of companion animals on suicidality (See Table A1). Outcomes were deemed inconclusive by this research team when the results did not provide a definitive interpretation as to how pets impacted suicidality. In the example of Palazzo et al. [47], an individual who engaged in a suicide–peticide (death by suicide which also involved the killing or attempted killing of pets) killed her dog prior to herself and left a note stating, “the dog is already sleeping … we will soon sleep together.” Due to the nature of postmortem analysis, it was unknown to the researchers how the relationship with her dog impacted her willingness to engage in suicidal behavior.

As mentioned above, another theme identified in the research was the existence of deaths by suicide, which also involved the killing or attempted killing of pets. Multiple research articles identified in this review contained forensic data analyzing suicide–peticide [47,48,49,50,51]. In most cases, the reason for killing a pet was unknown. Young et al. [51] analyzed media reports related to suicides involving pets and outlined categories to describe these instances: mercy killings (killing a pet due to concern for its welfare when the individual is gone), extended suicides (the understanding that the deceased human had a sense of extended self which included the pet animal), suicide pacts (where it was reported that multiple individuals or a family intended to end their lives together), and family annihilators (an individual takes the life of at least one family member in addition to their own).

## 4. Discussion

The overarching goal of this review was to collate the current literature in order to strategically determine what is currently known about the impacts of companion animals on suicidality, as well as to determine what gaps future studies should seek to address. The secondary goal of the review was to identify if the literature suggested companion animals to be a protective factor, a risk factor, or having no impact on suicidality. The results of this review revealed evidence of companion animals serving as protective factors against suicide, risk factors for suicide, and having no impact or inconclusive impact as outlined above.

Results suggest that there is a growing field of research on this topic, with 16 of 30 studies published between 2020 and 2024. This exponential growth highlights a recent interest in this topic; yet, additional research is warranted. Particularly, rigorous high-fidelity research with larger sample sizes is necessary to establish causality or to further understand the mechanisms by which companion animals may play a role in suicide prevention efforts. A majority of the research represented in this sample is qualitative. While qualitative data provide useful details in the initial understanding of a phenomenon and an individual’s lived experiences, there is a need for additional quantitative and mixed methods studies to strategically build upon the current evidence base.

More broadly, the need for standardized measures and manualized protocols throughout human–animal interaction research has been identified by Rodriguez et al. [52] and is demonstrated in the literature included in this review. Human–animal interaction researchers such as Herzog [53] have also suggested that studies on the “pet effect” have produced conflicting results, as is echoed in this review’s identification of companion animals contributing to both suicidality-related protective factors as well as risk factors. Further research is warranted to develop a comprehensive understanding of the impacts of companion animals on human well-being as it relates to suicidality.

Additionally, the existing research is primarily representative of the human relationship with dogs and cats and would benefit from understanding how additional species might impact suicide risk. The studies included in this sample also provided varying degrees of detail about participants’ relationships with animals. For example, some but not all studies reported on the length of pet ownership, or if the pet belonged to the study participant versus another member of that participant’s household. Collecting more detailed information about the bond between human and animal, as well as the characteristics of the relationship (i.e., if the pet lives in the home versus outdoors, length of pet ownership, frequency of contact with pet, etc.) can better inform understanding of nuanced ways in which pets influence suicide risk and allow for future comparative studies. Further, additional exploration is needed to understand the relationship between *T. gondii* exposure, pets, and suicide risk. Lastly, additional inquiry into the causes for suicide–peticide would help to inform preventive measures that can keep both humans and animals safe.

The limitations most commonly reported in the literature included sampling biases, small sample sizes, and lack of generalizable data. The articles represented various populations, yet despite the increased risk for suicide amongst military Veterans, only one article [54] specifically evaluated a veteran population. The need for additional research with Veterans and other high-risk populations is indicated.

### 4.1. Clinical Implications

The results identified in this scoping review underscore important clinical implications. This research team comprises multiple licensed mental health clinicians with suicide expertise who have developed the following recommendations based on the themes outlined above:

### 4.2. Incorporating Pets into Suicide Risk Assessments

The impact of pets on an individual’s suicidality is likely dependent on many factors, including the individual’s relationship with that specific pet. To best understand how an individual’s companion animal(s) impacts their suicide risk, it is advised to incorporate questioning and clinical assessment that seeks to understand the role a pet plays in that person’s life. As it is best practice to assess for other protective and risk factors (e.g., access to care; connectedness; prior suicide attempts), it is recommended to thoroughly identify how a person’s pet impacts their suicidality. For instance, if an individual with a history of suicidal behaviors has a strong bond with an aging or ill pet, this is an important consideration to explore when understanding chronic suicide risk. Even if the pet is alive, this information can be helpful in understanding the value of increased resources and interventions in preparation for the loss of that pet. A clinician may ask themselves, and their patient, how the loss of this pet may affect that individual’s likelihood of suicide. Alternatively, an individual may report that a pet is the primary protective factor keeping them alive. This should be respected and documented, as would other commonly identified protective factors such as social supports or child-related caregiving responsibilities. The development of specific questions focused on the role of pets may afford meaningful opportunities to better understand a person’s nuanced risk. Understanding each individual’s unique relationship with their pet, and how this relationship may impact suicidality, aligns with a client-centered approach to suicide prevention.

Observing an individual’s behavior as it relates to their pets may also provide insight into suicide risk. For example, if an individual at risk for suicide is making plans to re-home a beloved companion animal, this may be indicative of suicide planning. Similarly to an individual selling or giving away belongings, acquiring access to lethal means, or demonstrating other behaviors common in the development of a plan for suicide, it is necessary to evaluate the reason for which they wish to rehome their pet and explore the possibility that it is due to suicidal intent.

### 4.3. Incorporating Pets into Suicide Safety Plans

Another avenue for effectively incorporating pets into clinical practice and suicide prevention is by understanding the potential role of pets in suicide safety plans. As outlined above, pets can offer emotional support, distraction, social connection, caregiving responsibility, and other benefits to an individual experiencing suicidality. When developing a safety plan to reduce an individual’s risk of suicide, clinicians may consider including non-human companions. For example, daily dog walking may present a distraction from suicidal thoughts and an opportunity for mindfulness, or petting a beloved cat may contribute to emotion regulation. Pet keeping can also be a means for social connection and may provide avenues for engagement in life and community, which can be protective against suicide. Socializing at the local dog park or pet store may be incorporated into a suicide prevention plan as a means of purpose and/or connection.

### 4.4. Need for Resources

The themes outlined in this paper speak to the importance of pets in many people’s lives. In cases where pets serve as a protective factor against suicide, it is imperative to provide resources that allow humans and their pets to remain together. For example, pet-friendly housing, resources for pet care during inpatient or residential treatment, financial support for pet care, and more can contribute to companion animals serving as protective factors against suicide.

### 4.5. Strengths and Limitations

The use of rigorous PRISMA review methods and the diversity of expertise in the research team are strengths of this review. Following PRISMA guidelines ensures a rigorous and comprehensive search of the literature. Our research team’s diverse expertise ensures that multiple disciplines and perspectives were represented at every step of the process. Additionally, the combination of mental health practitioners and researchers ensures that findings are directly applicable to both research and practice. Though every effort was made to capture all relevant articles, it is possible that a relevant publication was missed. For example, due to the resources available, the exclusion of articles not published in English, Spanish, or French was a limitation of this review which may create a publication language bias and result in missed articles from diverse cultures. Further, the research team only utilized databases accessible to them without additional cost. Three articles were also excluded due to lack of access to the full text. The utilization of search terms specific to dogs and cats, but not other species (fish, horses, birds, etc.), is also identified as a limitation and an opportunity for improvement in future studies.

## 5. Conclusions

Suicide remains an international health epidemic that takes the lives of hundreds of thousands of people globally each year. The need for research focused on highly efficacious suicide prevention strategies is evident. Exploring the impact of pets on suicidality presents a creative and meaningful approach to understanding the psychosocial complexities of suicide risk. For many people, pets are considered family members or otherwise serve as key sources of social support. Incorporating pets into suicide risk assessments and prevention strategies may serve as a useful tool in preventing suicide. Additional rigorous research is needed to support this claim and fully understand this topic.

## Figures and Tables

**Table 1:** Study Participant Demographics.

Category	Sub-Category	*N* = Articles
Human Demographics		
Race/Ethnicity		
	Did not report	21
	Bi- or Multi-Racial	2
	White/Caucasian	6
	Native American	3
	Native Hawaiian/Pacific Islander	1
	Black	3
	Hispanic/Latino	3
	Middle Eastern	1
	Asian	2
	Other	1
Life Stage		
	Adult	21
	Elder	5
	Adolescent/Young Adult	3
	Did not report	2
Sex		
	Male	22
	Female	28
	Transgender Male	2
	Transgender Female	2
	Two-spirit	1
	Non-binary	4
	Other *	4
	Unknown/prefer not to say	1
Educational Level of Study		
	Did not report	26
	Some college education (with or without degree)	3
	High school (with or without diploma)	1
	Secondary school (grades 6–9)	2
	Primary/elementary (grades 1–5)	2
Mental Health Diagnoses		
	Did not report	13
	Personality disorder	2
	Depression	10
	Suicidal ideation or suicide attempts	8
	Anxiety disorder	6
	Bipolar disorder	2
	Schizophrenia or other psychotic disorder	4
	Obsessive/compulsive disorder	2
	Substance use disorder	1
	Post-traumatic stress	2
	Panic disorder or panic attacks	1
	Neurodivergence or autism spectrum disorder	2
	Attention deficit/hyperactivity disorder	1
	Eating disorder	1
	Paranoia	1
	Unspecified mental or physical health condition	2
Veteran Status		
	Did not report	29
	United States Veteran	1
Animal Demographics		
Species		
	Dog	24
	Cat	15
	Horse	7
	Other farm animal	3
	Reptile	5
	Amphibians	3
	Fish	9
Pet Ownership		
	Pet belonged to study participant	23
	Pet belonged to family member	3
	Pet belonged to other member of household	4
	Unclear or not reported	10

Footnote: * “other gender” was utilized to categorize studies in which participants did not identify as male or female, but also did not specify another gender identity (i.e., transgender, non-binary, etc.).

## Appendix A

**Table A1:** Selected Studies.

Year	First Author	Article Title	Journal	Published Language	Country	Study Design	Reported Impact of Companion Animals on Suicidality
2018	Hayden-Evans [35]	Pets provide meaning and purpose’: A qualitative study of pet ownership from the perspectives of people diagnosed with borderline personality disorder	Advances in Mental Health	English	Australia	Qualitative research	Protective Factor
2022	Schmitz [37]	Companion, friend, four-legged fluff ball’: The power of pets in the lives of LGBTQ+ young people experiencing homelessness	Sexualities	English	United States	Qualitative research	Protective Factor
2014	Hasegawa [48]	Assisted suicide and killing of a household pet: Pre-autopsy post-mortem imaging of a victim and a dog	Forensic Science, Medicine, and Pathology	English	Germany	Case report	Inconclusive or Unclear Impact
2012	Putney [42]	“Souldog”: The perceived impact of companion animals on older lesbian adults	UMI Dissertation Publishing/ProQuest	English	United States	Qualitative research	Inconclusive or Unclear Impact
2007	Fitzgerald [34]	“They gave me a reason to live”: The protective effects of companion animals on the suicidality of abused women	Humanity & Society	English	Canada	Qualitative research	Protective Factor
2023	Douglas [55]	Pet attachment and the interpersonal theory of suicide	Crisis: The Journal of Crisis Intervention and Suicide Prevention	English	United States	Cross-sectional study	Protective Factor; Risk Factor; No Impact
2022	El Frenn [56]	Association of the time spent on social media news with depression and suicidal ideation among a sample of Lebanese adults during the COVID-19 pandemic and the Lebanese economic crisis	Current Psychology	English	Lebanon	Cross-sectional study	Inconclusive or Unclear Impact
2009	Hagemeier [49]	Extended suicide using an atypical stud gun	Forensic Science International	English	Germany	Case report	Inconclusive or Unclear Impact
2022	Teimouri [46]	Prevalence and predictors of *Toxoplasma gondii* infection in psychiatric inpatients in Fars Province, Southern Iran	Frontiers in Psychiatry	English	Iran	Cross-sectional study	Inconclusive or Unclear Impact
2011	Akcan [39]	Unexpected suicide and irrational thinking in adolescence: A case report	Journal of Forensic and Legal Medicine	English	Turkey	Case report	Risk Factor
2024	Hawkins [33]	Young adults’ views on the mechanisms underpinning the impact of pets on symptoms of anxiety and depression	Frontiers in Psychiatry	English	United Kingdom	Qualitative research	Protective Factor
2015	Hutton [32]	Social provisions of the human-animal relationship amongst 30 people living with HIV in Australia	Anthrozoos	English	Australia	Qualitative research	Protective Factor
2023	Barcelos [41]	Dog owner mental health is associated with dog behavioural problems, dog care and dog-facilitated social interaction: a prospective cohort study	Scientific Reports	English	United Kingdom	Cohort study	Risk Factor
2015	Figueiredo [31]	Is it possible to overcome suicidal ideation and suicide attempts? A study of the elderly	Cien Saude Colet	Other	Brazil	Qualitative research	Protective Factor
1985	Helsing [57]	Dog and cat ownership among suicides and matched controls	American Journal of Public Health	English	United States	Cross-sectional study	No Impact
2014	Roseneil [58]	On meeting Linda: An intimate encounter with (not-)belonging in the current conjuncture	Psychoanalysis, Culture, and Society	English	United Kingdom	Case report	Inconclusive or Unclear Impact
2018	Sarlon [59]	Vegetative symptoms and behaviour of the therapy-accompanying dog of a chronically suicidal patient	BMJ Case Reports	English	Germany	Case report	Protective Factor
2021	Scanlon [60]	Homeless people and their dogs: Exploring the nature and impact of the human-companion animal bond	Anthrozoos	English	United Kingdom	Qualitative research	Inconclusive or Unclear Impact
2023	Smith [61]	Links between pet ownership and exercise on the mental health of veterinary professionals	Vet Record Open	English	United Kingdom	Cross-sectional study	No Impact
2021	Palazzo [47]	Integrated multidisciplinary approach in a case of occupation related planned complex suicide–peticide	Legal Medicine	English	Italy	Case report	Inconclusive or Unclear Impact
2013	Cooke [50]	Extended suicide with a pet	Journal of the American Academy of Psychiatry	English	United States	Case report	Inconclusive or Unclear Impact
2016	Hutton [30]	What am I going to do without him?’: Death anxiety, projection, and the human-animal relationship	Psychoanalytic Review	English	Australia	Case series	Protective Fator; Risk Factor
2020	Young [29]	A qualitative analysis of pets as suicide protection for older people	Anthrozoos	English	Australia	Qualitative research	Protective Factor; Risk Factor
2021	Love [28]	Best friends come in all breeds: The role of pets in suicidality	Anthrozoos	English	United States	Qualitative research	Protective Factor; Risk Factor; No Impact
2021	Hawkins [36]	I can’t give up when I have them to care for’: People’s experiences of pets and their mental health	Anthrozoos	English	United Kingdom	Qualitative research	Protective Factor; Risk Factor
2022	Mattock [40]	Discourses and silences: Pets in publicly accessible coroners’ reports of Australian suicides	Anthrozoos	English	Australia	Qualitative research	Risk Factor; No Impact
2023	Young [51]	Peticide: An analysis of online news media articles of human suicide involving pet animals	Anthrozoos	English	United Kingdom; United States; Australia	Qualitative research	Inconclusive or Unclear Impact
2021	Scarth [38]	Strategies to stay alive: Adaptive toolboxes for living well with suicidal behavior	International Journal of Environmental Research and Public Health	English	United States	Qualitative research	Protective Factor; Risk Factor
2019	Williamson [54]	An exploratory analysis of self-reported protective factors against self-harm in an enrolled veteran general mental health population	Military Medicine	English	United States	Cross-sectional study	Protective Factor
2021	Barcelos [27]	Understanding the impact of dog ownership on autistic adults: Implications for mental health and suicide prevention	Scientific Reports	English	United Kingdom	Qualitative research	Protective Factor
